# Plants of the Spontaneous Flora with Beneficial Action in the Management of Diabetes, Hepatic Disorders, and Cardiovascular Disease

**DOI:** 10.3390/plants10020216

**Published:** 2021-01-23

**Authors:** Maria Valentina Ignat, Teodora Emilia Coldea, Liana Claudia Salanță, Elena Mudura

**Affiliations:** 1 Department of Food Engineering, Faculty of Food Science and Technology, University of Agricultural Sciences and Veterinary Medicine, 400372 Cluj-Napoca, Romania; maria.socaci@usamvcluj.ro (M.V.I.); teodora.coldea@usamvcluj.ro (T.E.C.); 2 Department of Food Science, Faculty of Food Science and Technology, University of Agricultural Sciences and Veterinary Medicine, 400372 Cluj-Napoca, Romania; liana.salanta@usamvcluj.ro

**Keywords:** cardiovascular disease, chicory, dandelion, degenerative diseases, diabetes, mulberry, non-alcoholic steatohepatitis

## Abstract

The current pharmacological agents advised for the management of diabetes as well as cardiovascular and hepatic diseases are subject to numerous studies for safety and efficacy. Therefore, it is worth looking into alternative therapeutic aids such as natural products of medicinal plants. By a broad review of in vitro and in vivo studies on the various dandelion, chicory, and mulberry extracts, this work highlights their bioactive compounds and therapeutic action when used as a prevention and management aid in public health such as diabetes, cardiovascular disease, and hepatic disorders like non-alcoholic steatohepatitis. Natural products of dandelion leaves and root extracts can suppress the development of liver cancer, decrease insulin resistance, and suppress total triglyceride and cholesterol levels. Recent studies on mulberry leaves extracts indicated that they could decrease palmitic acid-induced lipotoxicity, increase total cholesterol and bile acid excretion, improve superoxide dismutase expression, and improve insulin resistance. Chicory root extracts boost satiety, reverse insulin resistance, and augment lipid metabolism thanks to their contents in chicoric acid, chlorogenic acid, and polysaccharides. *Taraxacum officinale* L., *Morus nigra* L., and *Cichorium intybus* L. present hepatoprotective, anti-inflammatory, antioxidant, hypolipidemic, and hypoglycemic activities and are shown to be advantageous in the management of obesity, dyslipidemia, Type 2 diabetes, and non-alcoholic fatty liver diseases. These plants are commonly available in the European spontaneous flora and more attention could be paid to their natural products.

## 1. Introduction

There is a need for finding further alternative solutions to public health concerns. The proposed resolution is to focus our attention on plant biomolecules and natural products, which can be used in the development of functional products for preventing and managing public health concerns such as diabetes, hepatic disorders, and cardiovascular disease [1,2,3,4,5]. Adding functional foods rich in beneficial compounds to the usual meals might represent a convenient solution for those who look for healthy nutrition without quitting food values, such as quality of ingredients, food safety, or organoleptic acceptance [6].

Type 2 diabetes mellitus (T2DM) and obesity are recognized as promoting factors of developing non-alcoholic fatty liver disease (NAFLD). The association between T2DM and NAFLD has been attributed to insulin resistance, which leads to hepatic lipid overflow [7,8]. Furthermore, NAFLD heightens mortality rates in association with cardiovascular disease (CVD), neoplastic processes, and hepatic failure [9].

The importance of finding alternative solutions for managing such medical conditions is easily seen in the light of their prevalence data. In 2017, the European Heart Network advised that yearly CVD leads to 3.9 million deaths in Europe and more than 1.8 million deaths in the European Union (EU). Moreover, CVD is accounted for 45% of all deaths in Europe and 37% of all deaths in the EU. Of all behavioral risk factors, it seems like dietary factors make the strongest impact regarding the risk of CVD mortality and CVD disability-adjusted life years at the population level across Europe. High systolic blood pressure is the most important medical risk factor. The incidence of diabetes in European populations is elevated as well and it has grown quickly over time, with more than 50% in several countries [10].

On a global level, approximately one out of 11 adults are diagnosed with diabetes mellitus (90% T2D) and, among the major risk factors, there are obesity, sedentary lifestyle, and the excessive consumption of red meat and processed meat, refined grains, and beverages containing added sugar [11]. Clinical trials have shown that diet and lifestyle modifications are effective in preventing T2DM in high-risk individuals. Although genetic predisposition can determine the predisposition to T2DM, unhealthful diets and inactive lifestyles are key factors of the present-day worldwide epidemic [12,13].

The incidence of NAFLD and non-alcoholic steatohepatitis (NASH) is the main cause of chronic hepatic illnesses in Western countries and this fact is becoming valid around the rest of the globe [14]. For example, the incidence of non-alcoholic fatty liver disease in Europe is about 24% [15], and, on a global level, is approximately 25% [16].

NAFLD is a hepatic expression of the metabolic syndrome and it usually develops along with insulin resistance, dyslipidemia, and obesity [17,18]. NAFLD is a clinical entity described by the presence of macrovesicular steatosis in ≥5% of hepatocytes in individuals who drink little or no alcohol and can be divided into two major subtypes. The first type is simple steatosis, which is the non-progressive type. The second one is non-alcoholic steatohepatitis, which is the progressive form that can further develop into cirrhosis and hepatocellular carcinoma [19].

The main recommendations in managing non-alcoholic steatohepatitis are lifestyle changes in what concerns diet and physical activity. Nevertheless, it was indicated that the bare behavioral changes in terms of diet habits and lifestyle rarely result in complete control or treatment of non-alcoholic hepatic disorders [20]. On the other hand, previously, the cessation of disease progression was noticed in patients diagnosed with non-alcoholic steatohepatitis who achieved weight loss through dietary changes and regular exercise. Complying with a hypocaloric and hypolipidemic diet and allocating 200 min per week of physical activity was shown to initiate the regression of hepatic fibrosis in patients with 10% or more bodyweight loss [21].

In the meantime, there are continuous assessments of pharmacological agents to evaluate their metabolic, anti-inflammatory, and antifibrotic actions. For example, in advanced clinical studies, drugs such as obeticholic acid demonstrated anti-fibrotic action in NAFLD and NASH. However, it was suggested that combinatorial therapies should have a better impact as etiology-independent therapies [22]. Moreover, in the past years, studies showed variable beneficial actions using obeticholic acid, elafibranor, and liraglutide-based therapies [23].

Upcoming studies are continuously investigating effective and safe pharmacological methods for the treatment of degenerative diseases. Meanwhile, designing and proposing functional beverages containing natural products of medicinal plants beneficial in managing diabetes, CVD, and NAFLD might help in improving the life quality and overall health status of patients.

Plants offer a variety of bioactive compounds that pose research interest for their beneficial properties in various health concerns. For example, plant-derived oligosaccharides and polysaccharides have favorable hypoglycemic action with no adverse side effects. Dietary carbohydrates comprising fiber are effective in managing hyperglycemia. Well-known oligosaccharides as well as xylooligosaccharide, isomaltooligosaccharide, fructooligosaccharide, galactooligosaccharide, and sucrose can help in managing glycemic and insulin metabolism [24].

Flavonoids are proven to be valuable in various public health concerns such as CVD, diabetes, and cancer. Anti-diabetic properties were attributed to shamimin, daidzein, epicatechin, myricetin, epigallocatechin, hesperidin, naringenin, hesperidin, chrysin, apigenin, genistein, kaempferol, luteolin, and quercetin [25]. Flavonoids, anthocyanidins, isoflavone flavonols, flavones, lignin, and proanthocyanidins have also been documented as beneficial in cardiovascular disease. It is as important to highlight that polyphenols have anti-hypertensive, atherogenic, and anti-inflammatory effects, and they can inhibit platelet aggregation and activation [26].

Although weight loss seems to be the main non-invasive solution in ameliorating non-alcoholic steatohepatitis [27], patients suffering from NAFLD and NASH may as well benefit from positive results by consuming products rich in bioactive compounds with proven anti-inflammatory and antioxidant activities. Such an example of an intensively studied beneficial compound is silymarin, which is a natural polyphenolic flavonoid of milk thistle (*Silybum marianum*) that addresses chronic inflammation diseases, insulin resistance, oxidative stress, hepatocellular protection, atherogenic dyslipidemia, and cardiovascular protection [28,29,30]. Moreover, *Geranium* species are as well documented to have antioxidant, hypoglycemic, and anti-inflammatory actions, which can be well applied in the management of diabetes, hepatic disorders, and cardiovascular disease [31,32].

In the following lines, we will briefly present studies conducted before 2010 and discuss some of the in vivo and in vitro studies published over the last 10 years regarding bioactive compounds and therapeutic potential of natural products of dandelion (*Taraxacum officinale* L.), mulberry (*Morus nigra* L.), and chicory (*Cichorium intybus* L.) in managing diseases such as diabetes, NASH, NAFLD, and CVD. These plants were selected for review because they are common to the spontaneous flora, constantly grow in Europe, are well resistant to climatic change, and survive in stressful weather conditions. Thus, these plants are sustainable plant sources of natural products [33,34,35]. Given these, more emphasis must be put on researching the health benefits of dandelion, chicory, and mulberry. These traditionally used plants should be further cultivated and harnessed into functional products using environmentally friendly technologies. In this sense, further studies and extraction techniques should be applied and assessed to obtain healthful products suitable for being tested in human subjects.

This review highlights the plant parts and some of their identified bioactive compounds with proven potential as being beneficial factors in preventing and managing diabetes, hepatic and cardiovascular diseases, and related complications, such as obesity, fibrosis, hyperlipidemia, and hyperglycemia. This review would allow researchers to bring their attention to these plants and their possible pharmacological and nutraceutical applications, which can aid the general effort of finding therapeutic methods into preventing and treating degenerative diseases.

## 2. Dandelion (*Taraxacum officinale* L.)—Brief Biochemical Profile and a Review of In Vivo and In Vitro Studies Evaluating Its Action in Managing Diabetes, Hepatic Disorders, and CVD

The genus *Taraxacum* is part of the *Asteraceae* family of the *Cichorioideae* subfamily, *Lactuceae* tribe. This plant’s geographical distribution is usually around the warm areas of the Northern Hemisphere and its users have been cherishing it for its curative properties since ancient times. Reports show that traditional medicine practitioners used *Taraxacum officinale* L. for treating dyspepsia, spleen, liver disorders, hepatitis, and anorexia [36]. Aqueous extracts of dandelion were also used traditionally through Asia, Europe, and North America for treating different types of cancer like leukemia and breast cancer, even if their working mechanisms were unknown [37,38,39].

The main reported components of dandelions, which consist of phenolic acids (chicoric, caffeic, and chlorogenic acids), terpenes (taraxacoside, ainslioside, taraxinic acid), and storage carbohydrates (inulin), which are thought to be accountable for the plant’s health-related properties [36,40,41].

The dandelion’s chemical composition of phenolic acids was studied in different types of extracts and determined by high-performance liquid chromatography (HPLC) identifying chlorogenic, caffeic, p-coumaric, sinapic, ferulic and chicoric acid, chicoric, and sinapic acid as the predominant compounds [42]. The biochemical analysis found chicoric acid as the major dandelion compound with a polyphenolic amount of 34.08 ± 1.65 g/kg in leaves and stems. Moreover, there are higher amounts of polyphenols in flowers and leaves than in stems. Dandelion’s roots are composed of carbohydrates (inulin- up to 45% of the total root compounds), carotenoids, fatty acids, minerals, sugars, choline vitamins, mucilage, and pectin [43]. The leaves and the plant’s flowers contain coumarins and numerous flavonoids [44].

Taraxasterol and taraxerol, which are identified in dandelion roots, have proven antitumor activities, anti-inflammatory, anti-histaminic, and antioxidant properties. It is suggested that these triterpenes are also beneficial in diseases such as Parkinson’s, Alzheimer’s disease, and diabetes [45]. Other compounds of dandelions, such as polyphenols, sesquiterpenes, and phytosterols are found beneficial in a variety of affections [43]. Taraxasterol was also proven to suppress liver cancer, at least partially. This is said to be obtained by intensifying the gene expression of Hint1 (Histidine Triad Nucleotide Binding Protein 1) to regulate Bax, Bcl2, and cyclin D1 expressions in a dose-dependent way [46].

The *Taraxacum* species including *T. officinale* contain chemical constituents such as sesquiterpenoids, triterpenoids, and phytosterols such as taraxasterol, arnidiol, faradiol, taraxacin, taraxinic acid, α-amyrin, β-amyrin, β-sitosterol, and stigmasterol. Flavonoids and phenolic acids such as quercetin, luteolin, hesperetin, apigenin, artemetin, isoetin, genkwanin, chrysoeriol, caffeic acid, chicoric acid, chlorogenic acid, ferulic acid, and p-coumaric acid. Other important chemical constituents like organic acids, inulin, and vitamins A, B, C, and D [47].

This study reviewed the dandelion leaf and root extracts and their benefits in managing diabetes, hepatic, and cardiovascular diseases. The dandelion’s leaves and roots appear to have a more significant anti-inflammatory, anti-cancer, and anti-microbial action than the dandelion’s flowers [48].

### 2.1. Dandelion Extracts and Their Natural Products Studied for Therapeutic Potential in Diabetes

As seen in Table 1, several extracts of whole dandelion plant were studied in vivo and in vitro settings. Moreover, a dandelion root extract in combination with other medicinal plants including chicory root and mulberry leaves was evaluated in vivo on non-obese diabetic mice. Some of the results suggested that dandelion extracts prevent diabetic complications, improve lipid metabolism, and present alpha-glucosidase inhibitory activity. However, some of the actual functional natural products of *T. officinale* responsible for the reported beneficial outcomes still had to be elucidated.

In 2012, dandelion leaves and roots were tested on streptozotocin (STZ)-induced diabetes in rats. The findings provided some evidence of hypoglycemic effects resulting after administering *T. officinale* leaves and roots of aqueous and ethanolic extraction. It was presented that the extracts, particularly the ethanolic extract, can enhance carbohydrate metabolism. It was also suggested that ethanolic extraction was more effective than the aqueous extraction and that the roots were more therapeutically effective than the foliage in managing and treating diabetes. The experimental findings also indicated that *T. officinale* extracts and their effects were dose-dependent. However, it was only hypothesized that the functional natural products responsible for this action might be dandelion’s root fructooligosaccharide, such as inulin [51].

Natural products and plant-derived compounds reveal anti-diabetic effects through mechanisms like inhibiting renal glucose reabsorption, reducing the activity of carbohydrate enzymes (α-amylase with β-galactosidase and α-glucosidase), lowering dietary blood sugar, and inhibiting potassium channel flow [43]. In this sense, an in vitro study conducted in 2015 on dandelion methanol and water extracts explored their α-amylase and α-glucosidase inhibitory actions and concluded that water and methanol extracts of the dandelion stem, roots, and flowers exhibit inhibitory activities powerful enough to consider the effective usage of this plant in managing diabetes [55]. The authors pointed out that the water extracts possessed the highest anti-diabetic properties than those of methanol extracts. In contrast, the stem extracts had the highest activity, followed by dandelion roots and flowers. Once again, the actual functional natural products accountable for the anti-diabetic action were thought to be dandelion’s fructooligosaccharides.

A review from 2015 noted that *T. officinale* exhibits therapeutic properties like those of black soybeans. In rat models of NAFLD treated with dandelion extracts, there was a substantial decrease of lipidic buildups in the liver, decreased hepatic tissue, and body weight, and a diminished serum cholesterol level. After administering dandelion leaf extracts, decreased insulin resistance was noted by activating the (5’ adenosine monophosphate-activated protein kinase) AMPK pathway. Additionally, the authors highlighted that polyphenols as well as flavonoids, and several other natural compounds have beneficial effects on NAFLD while preventing the emergence of some associated disorders. Natural products of dandelions can regulate the expression of several genes whose dysfunctions contribute to the buildup of lipid, fibrosis, swelling, oxidative stress, and insulin resistance [52].

T2DM is often met in connection with NASH and NAFLD. Since an increasing number of data shows that NAFLD elevates the risk of developing T2DM, it is presumed that NAFLD and NASH are specific clinical signs of T2D through a concurrent process of lipidic buildup, chronic inflammation, and hepatic fibrosis [57].

Recently, dandelion leaves and stem extracts were evaluated in vitro along with other medicinal plants for their anti-diabetic and anti-obesity properties and it was shown that the ethanolic extract of dandelion, roseroot, and water extracts of *Myrica gale* displayed substantial suppression of advanced glycation end-products formation (IC50 = 69.4, 74.0, 70.4 mg/L) in comparison to aminoguanidine (IC50 = 138 mg/L), which is the usual anti-glycation drug. It was concluded that the polyphenols of the previously mentioned plants are potentially useful in managing T2D and obesity [56].

**Figure 1:**
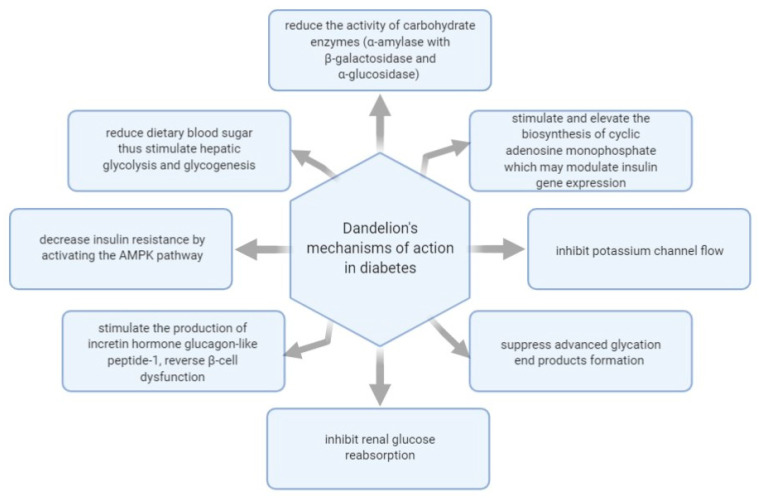
Mechanisms of action regarding dandelion’s effect in managing diabetes.

Figure 1 shows some of the mechanisms of actions investigated regarding *T. officinale*’s effect in managing diabetes.

### 2.2. Dandelion Extracts and Their Natural Products Studied for Therapeutic Potential in Hepatic Disorders

As seen in Table 2, dandelion extracts were investigated by several in vivo and in vitro studies regarding their beneficial therapeutic outcomes on a hepatic level. Among other findings, the results suggested that some of the dandelion’s natural products prevent hepatic necrotizing processes, modulate drug-metabolizing enzymes, inhibit tumor cell growth, and are beneficial in treating fatty liver hepatotoxicity caused by persistent alcohol consumption.

Hepatic damage can occur after prolonged administration of medications prescribed as part of specific pharmacotherapies. Such a case is APAP-induced hepatotoxicity, acetaminophen being an antipyretic and analgesic drug linked to several cases of hepatitis, cirrhosis, and liver transplants when its administration is prolonged or in overdose. In this sense, research was performed to evaluate dandelion’s capacity to diminish hepatic dysfunction induced by APAP through its natural antioxidant compounds. Thus, it was found that APAP orally dispensed to murine models at a dose of 200 mg/kg produced biochemical and histological lesions in the liver tissue. However, pre-treatment with hydroalcoholic extract of *Taraxacum officinale* L. leaves over the course of 10 days managed to prevent damage to the biochemical parameters produced by APAP, indicating a substantial hepatoprotective impact, most likely triggered by the extract’s content of phenolic compounds [59].

Over the course of 2010, several studies focused on various dandelion extracts’ therapeutic action in similar models of oxidative stress and hepatic damage caused by carbon tetrachloride (CCl_4_). In one of the studies regarding this topic, the extracts were administered for seven days, and hepatitis was induced by a single dose of CCl_4_ (50% CCl_4_/olive oil; 0.5 mL/kg bw) administration. Although CCl_4_ drastically raises serum AST and ALT activities, the pre-treatment with dandelion water extract significantly decreases the AST and ALT activities along with the hepatic lesions. It was suggested that the evaluated dandelion polysaccharides have hepatoprotective action by regulating inflammatory responses and oxidative stress [60].

During the same month of June 2010, another study was published on the hepatoprotective potential of two other dandelion root fractions, known as an ethanolic extract (ETO) and sesquiterpene lactones enriched fraction (SL), against CCL_4_-induced hepatotoxicity in mice. This time, the post-treatment with ETO and SL substantially attenuated hepatotoxicity, as it was shown by the reduced levels of hepatic enzyme markers, like ALT, AST, ALP, and total bilirubin. Finally, it was concluded that dandelion-sourced sesquiterpene lactones are protective against acute hepatotoxicity produced by CCl_4_ administration in mice [61].

The effectiveness of dandelion root hydroethanolic extract was evaluated in CCl_4_-induced hepatic fibrosis on male BALB/c mice. The mice were treated with CCl_4_ dissolved in olive oil (20%, *v*/*v*, 2 mL/kg) intraperitoneally (i.p.), twice/week for four weeks. The extract was administered i.p. once/day for the next 10 days, in doses of 200 and 600 mg/kg body weight. The results showed that the dandelion treatment reduced hepatic fibrinous deposits, reinstated histological architecture, and modulated the expression of glial fibrillary acidic protein and α-smooth muscle actin. It was suggested that the beneficial outcome of dandelion root hydroethanolic extract on CCl_4_-induced liver fibrosis is due to the inactivation of hepatic stellate cells and improved hepatic recovering abilities, which are actions possibly triggered by the extract’s content of chlorogenic acid or its other polyphenolic chemical constituents [62].

In September 2010, another study was published by a previously referenced research group, investigating the protective impact of a dandelion leaf aqueous extract (DLWE) in CCl_4_-induced hepatitis in Sprague-Dawley rats. The animals were divided into normal control, DLWE control, CCl_4_ control, and two DLWE groups (0.5 and 2 g/kg bw). After seven days of treatment, it was shown that the extract’s administration significantly decreased CCl_4_-induced AST and ALT activities in a dose-dependent manner, most likely due to the contents of luteolin, luteolin-7-O-glucoside, and polyphenols. The authors suggested that DLWE has a protective impact against CCl_4_-induced hepatic damage partially due to the decrease of oxidative stress and inflammatory processes resulting from cytochrome P450 activation by CCl_4_ [63].

Later, another *T. officinale* L. ethanolic leaf extract was studied compared to n-hexane dandelion leaf extract for its therapeutic action in CCl_4_-induced liver toxicity in rats. It was observed that the ethanolic leaf extract had a superior hepatoprotective action in CCl_4_-induced liver toxicity, possibly due to the presence of various phytochemicals (e.g., polyphenols, flavonoids), as it decreased the concentration of thiobarbituric acid reactive substances, hydrogen peroxide, and nitrite contents, which is usually over-increased by CCl_4_ toxicity [64].

Dandelion leaf extracts were also tested on acute liver damage induced by sodium dichromate intoxication in a murine model. It turned out that the leaf extract had a significant positive action on hepatotoxicity, oxidative stress, and genotoxicity. It was speculated that the hepatoprotective effect of dandelion leaf water extract results from its active compounds such as polyphenols, flavonoids, tannins, and ascorbic acid. As previously observed, it is thought that the therapeutic properties of dandelion leaf extracts in managing liver lesions result from their antioxidant compounds [65].

In 2017, two purified water-soluble polysaccharide fractions were isolated from dandelion root and tested for their hepatoprotective effects in a mouse model that mimicked APAP-stimulated liver damage in humans. The research showed that the two dandelion root polysaccharides composed of glucose, galactose, arabinose, rhamnose, and galacturonic acid can defend the liver from APAP-stimulated hepatic damage by activating the Nrf2-Keap1 pathway, which is the main regulator of cytoprotective responses to endogenous and exogenous stresses triggered by reactive oxygen species (ROS) and electrophiles [66].

On a hepatic level, the therapeutic properties of dandelions are also explored in lead poisoning. A group of researchers assessed the hepatic toxic actions of prenatal exposure to lead in rats and the probable protective action of dandelion-enriched diets. It was concluded that adding dandelions to pregnant and lactating rats’ diet protects offspring from lead poisoning, likely through the decrease of oxidative stress and hepatic damage. Moreover, it was shown that supplementing female rats’ diet with up to 2% dandelion extract does not provide a toxic effect, nor does it increase oxidative stress. It was finally assumed that the microsphere extract’s antioxidant action from the dandelion root would be responsible for improving the oxidative status during the lead poisoning [67].

Alcohol intake is a contributing factor to the onset of hepatitis and liver damage. Therefore, in 2010, the effects of *Taraxacum officinale* (dandelion) root against alcoholic liver damage were explored in HepG2/2E1 cells and Institute of Cancer Research mice. The hot water dandelion root extract managed to provide hepatoprotective action in the cells treated with ethanol, while the ethanolic root extract did not reveal compelling hepatoprotective action. The aqueous dandelion root extract, containing 2% of flavonoids and 0.013 mg/g of luteolin, ameliorated the malondialdehyde levels, indicating that it carries protective action in alcohol-induced liver toxicity by decreasing lipid peroxidation and increasing antioxidant potential [70].

In vitro and in vivo studies looked for a clear insight into the plant’s therapeutic properties in alleviating liver disorders when these are caused by improper nutrition. In this sense, a study was conducted on murine models of hepatic steatosis induced by a high-fat diet. It has shown that administering dandelion leaf extracts resulted in high antioxidant activity and an overall therapeutic action. More specifically, it was noted that the administration of *Taraxacum officinale* L. leaf extract along with the high-fat diet dramatically reduced hepatic lipid accumulation. Moreover, liver and body weight was higher in the model group where dandelion extracts were not administered along with the high-fat diet than those who had dandelion extracts added to their meals in concentrations of 2 g/kg and 5 g/kg. In addition, dandelion leaf extract suppressed the levels of triglycerides, TC, and insulin, as the authors suggested, most likely due to the extract’s contents of luteolin and chlorogenic acid [68]. Another in vivo experiment conducted by the same group of researchers on murine models suffering from hepatic steatosis induced by choline and methionine deficient diets further concluded that dandelion leaf extracts are hepatoprotective due to their antioxidant and anti-inflammatory actions, which may be triggered by the contents of luteolin and polyphenols [69].

In 2019, another study was published, which evaluated the effect of dandelion root extract on radiation-induced hepatic and testicular tissue injury. Male Wistar rats (WR) were exposed to 8.5 Gy of gamma radiation applied as a shot dose, and the extract (200 mg/ kg/day) was orally supplemented 14 days before and after irradiation. The findings indicated that administering dandelion root extract reduced oxidative stress in hepatic and testicular tissues, showing a substantial decrease in the levels of malondialdehyde and protein carbonyl with a noticeable growth in glutathione and the activity of superoxide dismutase (SOD), catalase (CAT), and glutathione peroxidase (GPx). These results were thought to be triggered by the extract’s contents of chlorogenic acid and taraxasterol. However, although the extract reduced histopathological alterations, it was suggested that the protective action was more significant in testicular tissue injury, adding that the extract should be better administered prior to radiation [74].

**Figure 2:**
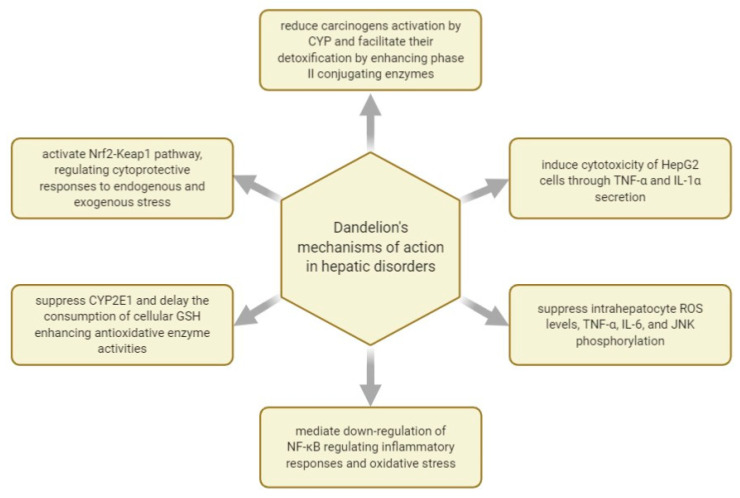
Mechanisms of action regarding dandelion’s effect in managing hepatic disorders.

Figure 2 shows some of the mechanisms of actions investigated with regard to *T. officinale*’s effect in managing hepatic disorders.

### 2.3. Dandelion Extracts and Their Natural Compounds Studied for Therapeutic Potential in Cardiovascular Diseases

As seen in Table 3, *T. officinale* extracts were evaluated regarding their activities on a cardiovascular level by several in vivo and in vitro studies. Thus, dandelion root ethanolic extracts and leafy vegetable mix of dandelion and other plants were shown as being effective in platelet anti-aggregating activity and in protecting cells from lipid peroxidation and oxidative DNA damage.

In 2010, a group of researchers published their results about investigating the hypolipidemic and antioxidative actions of dandelion roots and leaves in rabbits following a high-cholesterol diet. A total of 28 male rabbits were distributed into four smaller groups: the normal diet group, the high-cholesterol diet group, the high-cholesterol diet with 1% (*w/w*) dandelion leaf group, and the high-cholesterol diet with a 1% (*w/w*) dandelion root group. Following four weeks of treatment, the plasma antioxidant enzymes and lipid profiles were evaluated. The findings indicated that diets containing dandelion roots and foliage, containing phenolics like catechol, caffeic acid, ferulic acid, m-coumaric acid, p-coumaric acid, vanillic acid, and syringic acid, beneficially influenced plasma antioxidant enzyme activities and lipid profiles. Therefore, dandelion roots and leaves might protect against atherosclerosis related to increased oxidative stress [77].

The antioxidant properties of dandelion leaves and petals were also studied in vitro, in 2017, on the production of thiobarbituric acid reactive substances (TBARS, a lipid peroxidation marker) in human plasma. Four phenolic fractions from dandelion leaves and petals were studied, and they all had antioxidant properties since they suppressed lipid peroxidation, protein carbonylation, and protein thiols oxidation. It was also concluded that, in this case, petal fractions, containing the highest luteolin contents, had greater antioxidant activity than leaf fractions regardless of their concentration. The dandelion was finally indicated as being helpful for managing diseases associated with oxidative stress and with hemostasis alterations [80].

In 2018, phenolic fractions from dandelion leaves and petals (at the dose range of 1–50 µg/mL) were studied in vitro for their anti-platelet and antioxidant activities in blood platelets. It was noted that phenolic fractions extracted from dandelion leaves and petals varied in their anti-platelet, anticoagulant, and antioxidant activities due to the different chemical profiles. The four fractions were defined as phenolic-rich fractions (A and B, leaves and petals of 50%), a flavonoid-rich fraction (D, petals of 85%), and a mixed fraction (C, leaves of 85%). Fractions A and B, rich in cinnamic acid derivates, seemed more efficient in anti-platelet and antioxidant action than flavonoid-rich fractions (fractions C and D). The main phenolic acid in fractions A, B, and C consisted of L-chicoric acid. The study finally demonstrated the anticoagulant activity of dandelion leaf and petal extracts and suggested that the anticoagulant actions of the analyzed phenolic fractions are correlated with a modulation of thrombin activity due to its suppression. Moreover, it was concluded that the dandelion leaf and petal extracts of 50% fractions offer natural compounds beneficial in preventing and treating cardiovascular diseases through antioxidant, anti-platelet, and anticoagulant actions [81].

Even though many of the dandelion root’s potentially bioactive components have been already discussed, such as hydroxycinnamic acids (HCAs) and sesquiterpene lactones (SLs), new compounds are being revealed. In an in vitro study, five dandelion root formulations (A–E) with different chemical contents were evaluated. About 100 phytochemicals were identified, comprising novel compounds for the genus *Taraxacum* and the plant kingdom, like amino acid-SL adducts. Moreover, none of the dandelion root extracts triggered blood platelet lysis (tested range of 0.5–50 μg/mL). It was indicated that dandelion roots are a secure and effective source of a diverse class of natural compounds holding antioxidant, anticoagulant, and anti-platelet properties [82].

In 2019, an in vitro study examined five dandelion root fractions with different chemical profiles for modifications produced in the human platelets’ model using selected hemostatic parameters. The greatest anti-platelet prospective was displayed by the formulation supplemented with hydroxyphenylacaetate inositol esters—PIEs (fraction C). Nevertheless, the greatest overall protective action against lipids and proteins oxidation of platelets, and the top suppressing action on the production of superoxide anion, were noticed using fraction A (SL-amino acid adducts enriched fraction). Thus, it was once again suggested that extracted biomolecules of the dandelion root can be regarded as suitable natural products to be employed in preventing and treating cardiovascular diseases linked to blood platelets hyperactivation [83].

The purpose of another in vitro research was to assess the action of dandelion’s chicoric acid on the biological profile of human plasma and blood platelets. Four phenolic fractions, acquired from leaves (fraction A and B) and petals (fraction C and D), with different concentrations of chicoric acid and L-chicoric acid extracted from dandelion foliage, were studied on biomarkers of oxidative stress, coagulation parameters, and blood platelet activation. The results suggested that chicoric acid extracted from dandelion leaves and petals offers antioxidant and anti-adhesive potential without cytotoxicity [40].

In 2020, phenolic extracts of dandelion leaves and petals were investigated as potential regulators of antioxidant and lipid profiles in male WR. The animals were supplemented over the course of four weeks with *T. officinale* extracts (694 mg/kg of diet = 11.9 ± 0.6 mg daily). The dandelion leaf and petal extracts assured a dose of ± 0.05 and 1.41 ± 0.07 mg l-chicoric acid per day and have demonstrated antioxidant actions, noted as diminished levels of thiobarbituric acid-reactive constituents, in spleen (≈0.8-fold, leaves and petals), brain (0.53-fold, leaves), and thoracic arteries (0.59-fold, petals). Moreover, dandelion leaf extracts impacted the lipid profile by diminishing triglycerides (0.44-fold), TC (0.73-fold), a lipoprotein combined index (0.32-fold), and a plasma atherogenic index (0.62-fold). Finally, it was suggested that dandelion leaf and petal phenolic fractions containing high contents of l-chicoric acid are valuable plant materials with potential beneficial antioxidant actions on a cardiovascular level [78].

Another recent study reported the phytochemical profile of dandelion fruit extract and assessed its antiradical, antiplatelet, and antioxidant characteristics on a model of hemostasis. Thus, it was shown that the main flavonoids and phenolic acids of dandelion fruits consist of luteolin and L-chicoric acid compounds, which might be employed in cardiovascular disease therapies [84].

**Figure 3:**
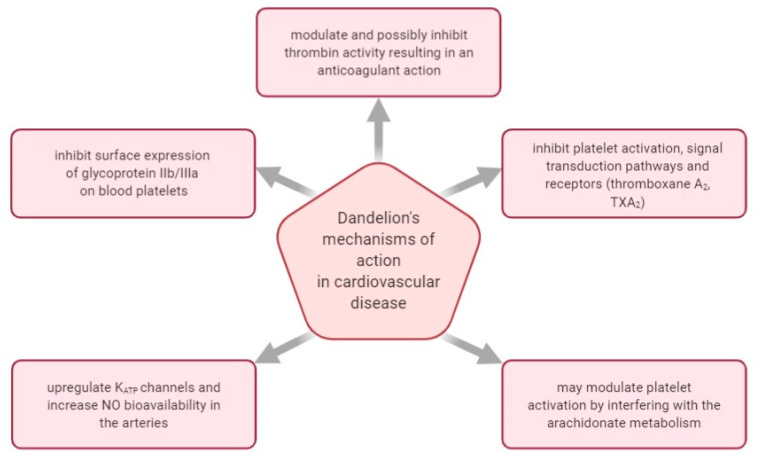
Mechanisms of action regarding dandelion’s effect for managing cardiovascular disease.

Figure 3 shows some of the mechanisms of actions investigated regarding *T. officinale*’s effect in managing cardiovascular disease.

## 3. Black Mulberry (*Morus nigra* L.)—Brief Biochemical Profile and a Review of In Vivo and In Vitro Studies Evaluating Its Action in Managing Diabetes, Hepatic Disorders, and CVD

Roughly 100 species of *Morus* have been described, such as *Morus alba* (white mulberry), *Morus rubra* (red mulberry), and *Morus nigra* (black mulberry). The mulberry plant is monoecious or dioecious, reaching up to 10–12 m in height. *Morus nigra* (*Moraceae* family) is commonly distributed in Asia, Africa, Europe, and America [85]. Given its therapeutic properties, the leaves, root barks, branches, and fruits are traditionally used in medicinal preparations to manage diabetes mellitus, atherosclerosis, hyperlipidemia, and hypertension [86]. Various extracts of mulberry leaves were studied over the last 10 years, investigating their bioactive compounds’ pharmaceutical in public health concerns, such as diabetes, hepatic diseases, and cardiovascular diseases.

In 2012, the leaves of *M. nigra, M. alba*, and *M. rubra* were studied to highlight their antioxidant components and scavenging potential. Analyzing these three species’ proximal composition revealed that the lower ash values were found in *Morus alba* while the maximum ash values were reported in *M. rubra* extracts. *M. nigra* presented the highest moisture and fiber content when compared to *M. alba* and *M. rubra*. Nevertheless, *M. alba* had the highest lipid composition and *M. rubra* had the highest protein content. The highest total flavonoid content was determined in the *Morus nigra* leaf extract while *M. rubra* had an overall superior total flavonoids content. Ascorbic acid was found primarily in *M. alba*, which was followed by *M. nigra* and *M. rubra*. It was finally suggested that *Morus nigra* would be more suitable for disease prevention than the other two species due to its superior phenolics content of 2,2-diphenyl-1-picrylhydrazyl (DPPH) and 2,2’-azino-bis(3-ethylbenzothiazoline-6-sulfonic acid (ABTS) radical scavenging capability [87].

Another study compared *M. alba* and *M. nigra* leaves using non-targeted UHPLC-MS and concluded that both species present high potential for pharmaceutical applications. The phenolic fractions of black mulberry leaves present mainly (poly)phenolic compounds, with flavonols being the main group of flavonoids having concentrations varying from 3.7 to 9.8 mg/g dry weight in Spanish clones. Caffeoylquinic acids and a few simple acids were also detected together with organic acids. Overall, 20 flavonols were identified as glycosylated forms of quercetin and kaempferol. There were more derivates of quercetin than of kaempferol and isoquercitrin along with quercetin-malonyl-glucoside that were reported among the major individual flavonoids of mulberry foliage [88].

In 2019, the phytochemical examination of a hexane phase from black mulberry leaves revealed steroid β-sitosterol, while the ethyl acetate phase highlighted two glycosylated flavonoids, kaempferol-3-O-glycoside, and quercetin-3-Oglycoside. Their findings, too, showed that this plant species of the spontaneous flora is a good flavonoids source. Therefore, it should be further characterized for its pharmacological properties regarding antidiabetic, antioxidant, anti-inflammatory, and antihyperlipidemic activities [89].

Although this review paper covers the natural products and therapeutic properties of black mulberry leaves, it is worth mentioning that black mulberry fruits contain different levels of phenolic compounds consisting of gallic acid, catechin, chlorogenic acid, caffeic acid, syringic acid, *p*-coumaric acid, ferulic acid, *o*-coumaric acid, vanillic acid, rutin, and quercetin, with chlorogenic acid and rutin being the core phenolics. Organic acids, sugars, vitamin C, antioxidant activity, and phenolics vary substantially in white and black mulberry genotypes, and the highest antioxidant action was measured predominantly in black mulberry genotypes [90,91,92,93].

### 3.1. Mulberry Extracts and Their Natural Products Studied for Therapeutic Potential in Diabetes

Before 2010, the effect of black mulberry leaf extracts on diabetes was studied by two in vitro studies and in two in vivo settings. However, more studies were published after 2010. As seen in Table 4, the extracts are accounted for reducing diabetic symptoms, neuropathy, hyperglycemia, and inhibiting the carbohydrate metabolism enzyme activities.

Diabetes mellitus is a chronic disease defined by the overproduction of ROS, which affects the lysosomal membrane integrity, and, as a metabolic disorder, diabetes is also described by enzymatic alterations [106]. In 2015, a study investigated the catalytic characters and gene expression of hepatic arylsulfatase B (ASB) in streptozotocin-induced diabetic rats treated with black mulberry leaf extract. The results indicated that the black mulberry leaf and fruit extract administered as a powder managed to suppress the blood sugar levels in diabetic rats and substantially diminished hepatic ASB activity. The study’s published data revealed that the daily supplementation of *M. nigra* foliage has a beneficial action in the correction of diabetes-stimulated ASB alteration [96].

Moreover, about four years before the above study, an aqueous black mulberry leaf extract (Botucatu, São Paulo State, Brazil) was evaluated for its effects over maternal reproductive results, lipid and oxidative stress outline, and fetal anomaly prevalence when orally administered to streptozotocin-induced diabetic and non-diabetic WR. The study concluded that administering a dose of 400 mg/kg/day of black mulberry leaf extract, by gavage, from day 0 until the day 20 of pregnancy resulted in an antioxidant outcome contributive to a reduced occurrence of skeletal and visceral abnormalities in progenies from diabetic dams. However, it could not regulate maternal hyperglycemia, pregnancy rate, and placental–fetal development in diabetic rats [97].

Another study evaluated an ethanolic mulberry leaf extract’s properties in diabetes models induced by a high-fat diet and injection of 35 mg/kg STZ in male WR. The extract was administered to one of the four groups over the curse of four weeks. Kidney and blood samples were subjected to biochemical analysis such as fasting blood glucose level, albumin, creatinine, urea and uric acid concentrations, white blood cells, hemoglobin, hematocrit, and histological assessment. The results showed that fasting blood glucose, creatinine, urea, and uric acid displayed considerably lower levels in the extract-treated group than the group lacking mulberry leaf treatment. Moreover, histology evaluation proved that glycogen accumulation, fatty degeneration, and lymphocyte infiltration in the extract-treated group were only mild while they were moderate in the non-treated group [98]. These results were associated with the contents of mulberry leaves in 1-deoxynojirimycin (1- DNJ), quercetin-3-O-βD-glucopyranoside, phytoalexins moracin C, moracin N, and chalcomoracin. It was also indicated that mulberry leaf extracts, such as mulberry leaves tea, can be addressed to hyperuricemia and nephropathy in diabetes. Diabetic nephropathy results from overly increased oxidative stress, and mulberry leaves contain antioxidant components such as 1-DNJ, flavonoids, polyphenols, and polysaccharides with beneficial therapeutic actions [99].

To explain the regulatory mechanism of mulberry in treating diabetes, in 2011, a study evaluated 1-DNJ and polysaccharides extracted from mulberry leaves on alloxan-induced diabetic mice. Administering a daily oral treatment with polysaccharides (150 mg/kg body weight) to diabetic mice for three months resulted in postprandial blood glucose reduction and lessened toxicity caused by prolonged supra-physiological glucose to pancreatic cells. Moreover, it was concluded that polysaccharides might control hepatic glucose metabolism and gluconeogenesis by up/down-regulating the expression of rate-limiting enzymes (glucokinase, phosphoenolpyruvate carboxykinase, and glucose-6-phosphatase) in the liver and up-regulating the pancreatic and duodenal homeobox factor-1 (PDX-1), insulin-1, and insulin-2 expressions in the pancreas [100].

Mulberry foliage extract enriched with deoxynojirimycin (DNJ) was studied on postprandial hyperglycemia in patients with impaired glucose metabolism. After three months of supplementation with *M. nigra* extract (6 mg DNJ, t.i.d.) to 76 subjects with fasting plasma glucose varying between 110–140 mg/dL, it was determined that prolonged ingestion of *M. nigra* leaf extract with high DNJ contents can result in enhanced postprandial glycemic control, especially in cases of impaired glucose metabolism [95].

Another research investigated the coincubation of 0.1–10 μmol/L DNJ with mature 3T3-L1 adipocytes and established that the genes/proteins expression of insulin receptor, phosphatidylinositol-3-kinase, protein kinase B, and adiponectin were amplified in a dose-dependent manner. However, it was concluded that further studies are needed to clarify the molecular mechanism of action [107].

In 2020, there was another attempt to assess the mechanism of action and bioactivity of mulberry foliage polyphenols in preventing T2D by inhibiting disaccharidase and glucose transport in Caco-2 cells. The mulberry leaves extract’s main composition consisted of chlorogenic acid, rutin, benzoic acid, and hyperoside. The results indicated that mulberry leaf polyphenols hindered glucose absorption by curbing the sodium-dependent glucose cotransporter-1–glucose transporter 2 pathway by downregulating phospholipase mRNA expression, protein kinase A, and protein kinase C [105].

The leaves of black mulberry were studied compared to the leaves of *Bauhinia variegate* L., which is a species of flowering plant in the legume family *Fabaceae*. The study evaluated the impact of administering these plants’ extracts orally on hyperglycemia, insulin activity, and renal and hepatic function in an STZ-induced diabetic murine model. The HPLC with a diode-array detection analysis of the extracts highlighted major flavonoids being rutin, quercitrin, quercetin, and hesperidin, and phenolic acids as chlorogenic and p-coumaric acids. Quercitrin was found as the key flavonoid in *M. nigra* foliage extract (2.75 mg/g), and rutin was the main one in *B. variegate* extract (4.38 mg/g). Given the elevated insulin serum levels measured in the rats treated, it was suggested that the black mulberry leaf extract might be boosting the endogenous insulin secretion. Moreover, it was advised that the amount of elevated polyphenols identified in *M. nigra* foliage extract might add to its antioxidant action in protecting the liver and kidneys from tissue injury linked to hyperglycemia [101].

In January 2020, a group of researchers explored a hexane fraction of black mulberry Brazilian foliage (Hex-Mn) and their action on carbohydrate digestion and absorption in diabetic mice. HPLC analysis revealed the presence of flavonoids isoquercitrin and kaempferol-3-O-rhamnoside. It was shown that administering Hex-Mn through an oral route to diabetic mice led to the delay of carbohydrate digestion but not to the glucose transport through a brush border membrane of the intestine, which contributed to the reduction of postprandial hyperglycemia. Moreover, it was suggested that Hex-Mn was more effective in suppressing the α-glucosidase enzyme than the a-amylase activity in vitro [102]. Similarly, a later study concluded that the black mulberries leaf extract obtained through maceration acts as an α-glucosidase inhibitor. Their *M. nigra* extract inhibited the α-glucosidase enzyme with an IC_50_ value of 549.7 μg/mL [108].

**Figure 4:**
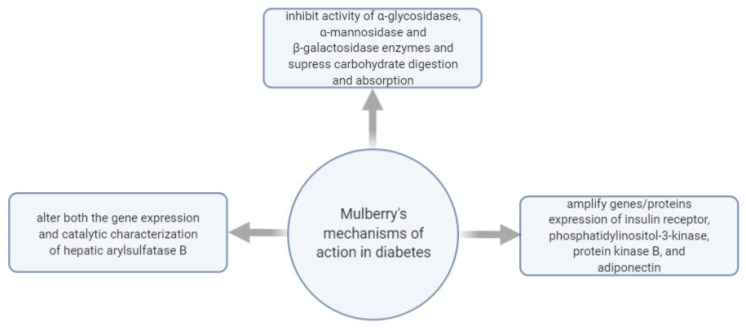
Mechanisms of action regarding mulberry’s effect in managing diabetes.

Figure 4 shows some of the mechanisms of actions investigated regarding *M. nigra*’s effect in managing diabetes.

### 3.2. Mulberry Extracts and Their Natural Products Studied for Therapeutic Potential in Hepatic Disorders

After checking the literature published before 2010, it was found that, before 2010, there are no relevant studies regarding black mulberry extracts and their potential benefits in treating or preventing hepatic diseases. However, in Table 5, there are summaries of the later studies conducted to assess mulberry’s action in managing hepatic disorders.

As in the case of dandelion leaf extracts, black mulberry leaves have a potentially beneficial and partly proven impact in cases of hepatic lesions induced by excessive or chronic drug administration. In 2014, an MeOH- H_2_O extract of *M. nigra* foliage was dispensed in doses of 250 mg/kg and 500 mg/kg through an oral route to mice suffering from paracetamol-induced hepatic damage, and it resulted in lower hepatic enzymes and total bilirubin levels. These hepatoprotective properties were attributed to the high contents of quercetin, luteolin, and isorhamnetin [109].

The antioxidant actions of *Morus* leaf ethanolic extracts were also examined in other cases of drug-induced hepatotoxicity. In vivo and in vitro studies were performed on methotrexate-induced hepatotoxicity models of male rats and on human HepG2 cells. The results indicated that the administration of *M. nigra* leaves extract is advised simultaneously with the anti-rheumatic drug therapy given the extract’s antioxidant and cytoprotective characteristics, which likely resulted from the plant’s flavonoid content [85].

Black mulberry leaves and pulp were also evaluated in what concerns their impact on the glycemic response and redox profile on a hepatic level in alloxan-induced diabetic rats. The findings indicated that the hydroalcoholic foliage extracts diminished the SOD-CAT ratio and carbonylated proteins levels by decreasing oxidative stress. Finally, it was suggested that black mulberry leaves could be used to control hyperglycemia as they increased the serum insulin level and present hepatoprotective action given the attenuated liver damage markers [110]. The authors concluded as well that the black mulberry leaves extract contained more phenolic compounds than the fruit extract, and that the therapeutic results are most likely triggered by caffeoylquinic acid contents, which were identified in the leaves but not in the fruits.

The effect of lyophilized black mulberry leaf extracts on CCI_4_-induced hepatic injury in a rat model was investigated. The leaf extracts at doses ranging from 150 to 300 mg/kg were administered to rats by i.p. injection for eight weeks. Black mulberry extracts counteracted protein oxidation caused by CCI_4_ and exhibited the capability to control the activity of SOD and GPx. Moreover, the extracts counteracted the CCI_4_-induced rise in AST and gamma-glutamyl transferase levels. Finally, leaf extracts offered substantial protection from CCl_4_-induced hepatic injury, and it was suggested that they might also represent a novel approach for treating hepatic diseases [111].

In what concerns the *M. nigra*’s toxicity, a study published in 2018 aimed to identify the chemical profile of EtOH leaves extract and perform a toxicological study in rats of both genders. In the acute exposure group, 2000 mg/kg of the extract was orally administered, and signs of toxicity and mortality were observed. The extract was orally dispensed for 28 days in doses of 500, 750, and 1000 mg/kg in the sub-acute exposure group. The extract’s chemical profile was determined through HPLC/DAD analysis and the findings indicated that quercetin and caffeic acid were the main compounds. After evaluating its action following acute administration, the EtOH *M. nigra* leaf extract was graded as a safe product (category 5), according to the protocol. Moreover, in the subacute exposure group, the researchers noted a reduction in AST in males (750 and 1000 mg/kg) and females (1000 mg/kg) and a decrease of TC in females (750 and 1000 mg/kg). Finally, the ethanolic *M. nigra* leaf extracts were marked as safe when administered orally. They exhibited protective action of organs along with cholesterol-lowering actions, likely due to the extract’s content of quercetin and caffeic acid, which are known as the mulberry leaf’s major compounds [112].

A recent study attempted to assess the mechanism through which the MeOH extract of black mulberry foliage curbs the damage caused by APAP in various murine tissues, including hepatic tissues. APAP (500 mg/kg) was administered orally with or without black mulberry leaf extract (150, 300, and 500 mg/kg) over the course of four days. It was, thus, indicated that the crude extract had powerful antioxidant activity (EC50 = 42.97 µg extract/mL) given the significant contents of polyphenols and flavonoids, such as gallic acid, chlorogenic acid, catechin, and rutin. Furthermore, the initially-modified levels of glutathione S transferase activity, lipid peroxidation, and liver and kidney functions were radically overturned when black mulberry MeOH extract was administered to the APAP group. Finally, it was indicated that *M. nigra* leaves’ natural products including potential candidates for genetic protection and the treatment of organotoxicity was caused by heightened oxidative stress [113].

**Figure 5:**
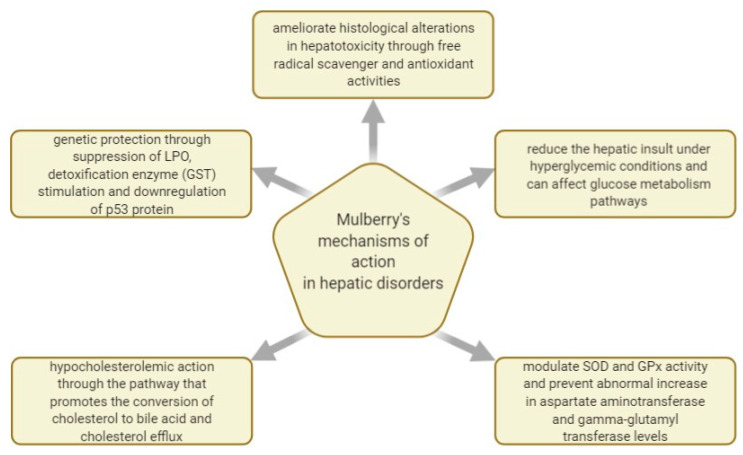
Mechanisms of action regarding mulberry’s effect in managing hepatic disorders.

Figure 5 shows some of the mechanisms of actions investigated regarding *M. nigra*’s effect in managing hepatic disorders.

### 3.3. Mulberry Extracts and Their Natural Products Evaluated for Therapeutic Potential in Cardiovascular Disease

After checking the literature published before 2010, it was found that there are no relevant studies regarding *M. nigra* leaf extracts and their potential benefits in treating or preventing cardiovascular disorders. However, in Table 6, there are some summaries of the later studies conducted to assess mulberry’s action in managing cardiovascular disease.

Black mulberry leaves were traditionally used to remedy ailments connected with various hepatic disorders and heart diseases. Currently, their extracts are studied for pharmacological and nutraceutical applications due to the reported bioactive compounds. Over the last 10 years, a few in vivo and in vitro studies were published on the *M. nigra*’s leaves therapeutic properties on a cardiovascular level, attributed mainly to its antioxidant properties.

In 2016, a black mulberry leaf extract at a dose of 200 mg/kg combined with binahong (*Anredera cordifolia* [Ten.] Stennis) leaf extract was administered to hyperlipidemic-induced rats for 21 days. Powdered crude herb of binahong and mulberry leaves were extracted by ethanol 70% and ethanol 95%, following the reflux method for 3 h with three repetitions. The combination of extracts showed superior effectiveness in a dose-dependent manner than that of the reference drug simvastatin in reducing TC and LDL serum levels [114].

Two black mulberry leaves’ aqueous solutions obtained through infusion and decoction, and a hydromethanolic extract of black mulberry leaves was compared to fenofibrate-based therapy to highlight their hypolipidemic abilities. Fenofibrate treatment is usually recommended for lowering cholesterol and for preventing cardiovascular complications. However, in what concerns the extracts, it was determined that the infusion extract of black mulberry leaves had superior contents of antioxidant polyphenols like chlorogenic acid and quercetin, while decoction extract offered a greater ascorbic acid content. The hyperlipidemic rats were treated with 100, 200, or 400 mg/kg of black mulberry extracts and showed diminished serum cholesterol, triglycerides, and regulated lipoproteins. Moreover, black mulberry leaves infusion reduced lipid peroxidation in the liver, kidney, and brain. It also resulted in a stronger hypolipidemic effect where high-density lipoprotein (HDL) improved, and LDL dropped. Finally, the potential therapeutic effect attributed to the high contents of quercetin and chlorogenic acid of the black mulberry leaf infusion was indicated as beneficial in dyslipidemia and related oxidative stress [115].

Although some studies are exploring the action of white mulberry leaves and black mulberry fruits over triggering conditions of cardiovascular diseases, obesity, and diabetes [117,118], there is very little preclinical information given over the last 20 years on the therapeutic effects of black mulberry leaves on cardiovascular disease, with the fruits being mainly studied for their content of anthocyanins, proven to be effective in managing cardiovascular disease and triggering factors such as hyperlipidemia [119,120].

As mentioned in the previous chapters, cardiovascular ailments are often triggered by diabetes and obesity. In these regards, a study published in 2020 investigated the impact and the action mechanism of dietary black mulberry leaf powder on fat deposition in fattening pigs. The animals were randomly allotted to a normal diet or a 5% (*w/w*) mulberry leaf supplemented diet. Changes in backfat thickness were assessed with blood triglycerides, cholesterol, serum hormones, and leptin-related signaling activity. The mulberry leaf supplemented diet reduced serum triglycerides and free cholesterol concentrations while it increased the ratio of HDL to LDL. Finally, it was indicated that administering black mulberry leaves as diet supplements in fattening pigs have potent obesity preventive action, offering a promising start for developing mulberry leaves-based products as anti-obesity agents [116].

*M. nigra* leaf extract’s therapeutic action was also studied on symptoms and quality of life among climacteric women. Women go through various hormonal modifications during the menopausal transition that heightens the probability of developing certain diseases and deteriorate women’s quality of life. This study is included in this chapter given that the most frequently met symptoms of hormonal changes also comprise cardiovascular affections among palpitations, headaches, depression, irritability, and fatigue. Furthermore, 250 mg of black mulberry leaf powder, 1 mg of estradiol, or placebo were administered for 60 days to 62 climacteric women. The conclusion was that climacteric symptoms and the women’s quality of life improved after administrating *M. nigra* leaf powder, similarly to the estradiol outcome [121].

**Figure 6:**
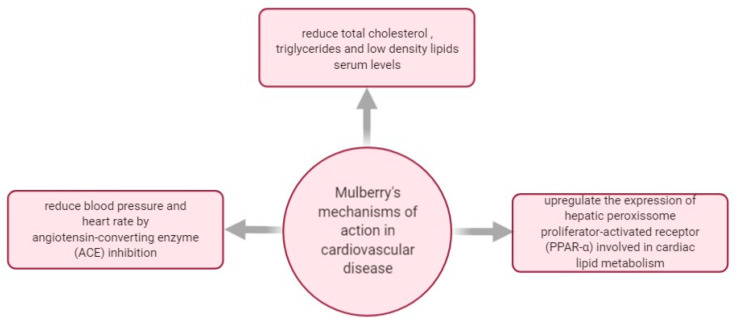
Mechanisms of action regarding mulberry’s effect in managing cardiovascular disease.

In Figure 6, there are some mechanisms of actions investigated regarding *M. nigra*’s effect in managing cardiovascular disease.

## 4. Chicory (*Cichorium intybus* L.)—Brief Biochemical Profile and a Review of In Vivo and In Vitro Studies Evaluating Its Action in Managing Diabetes, Hepatic DISORDERS, and CVD

*Cichorium intybus* L., commonly named chicory, is a perennial herbal plant of the dandelion family *Asteraceae*, mainly seen with bright blue flowers, and seldom pink or white [122]. Generally distributed in Asia and Europe, all the plants’ parts were traditionally used in medicinal preparations due to their considerable contents of antioxidant phytochemicals thought to have a beneficial impact in preventing and treating various illnesses such as fever, diarrhea, jaundice, and gallstones [123,124]. Different chicory types are grown for their salad leaves, chicons, or roots, and regularly used for inulin extraction, for preparing coffee substitutes, or for feeding livestock [125,126].

In 2011, the *C. intybus* root and peel ethanolic extracts were reported to contain rich contents of inulin (60.1 g and 46.8 g per 100 g of fresh mass), with a polymerization degree ranging between 3 and 10 while phenolics like caffeoylquinic acids consisted in 0.5 and 1.7 g per 100 g of fresh mass. Ethanolic extracts of leaves and seeds marked particularly lower inulin mass fractions (1.7 and 3.2 g per 100 g of fresh mass) and greater phenolics mass fractions (9.6 and 4.22 g per 100 g of fresh mass) like caffeoylquinic acids, chicoric acid, and quercetin glucuronide [127].

Alkaloids, inulin, sesquiterpene lactones, coumarins, vitamins, chlorophyll pigments, unsaturated sterols, flavonoids, saponins, and tannins are among the medicinally important compounds of chicory. Chicoric acid was identified as the main compound, while the plant’s main fraction consists of aliphatic compounds and their derivatives. The minor fraction is represented by terpenoids [124,128].

It was further validated that the chicory plant contains important phytochemical compounds such as carbohydrates, alkaloids, glycosides, phytosterols, amino acids, phenols, flavonoids, fixed oil, and fats [129]. Given this, in the last 10 years, several studies investigated the plant’s potential to be used in pharmaceutical applications. These would cover various ailments such as hepatic diseases, diabetes, and cardiovascular disease. Relevant in vivo and in vitro studies highlight the application of chicory extracts and their natural products employed in therapeutic actions.

### 4.1. Chicory Extracts and Their Natural Products Studied for Therapeutic Potential in Diabetes

After checking the literature published before 2010, it resulted that there are no relevant studies regarding *C. intybus* roots extracts and their potential benefits in managing diabetes. However, it was indicated that the whole chicory plant extract possesses hypoglycemic and hypolipidemic properties.

Table 7 summarizes some of the later studies conducted to assess the chicory’s action in managing diabetes.

Inulin-type fructans extracted from chicory, Jerusalem artichoke, and blue agave have been tested in diabetic and non-diabetic models for their effect on body weight, blood metabolites, and fecal bacteria. The results demonstrated that fructans could modulate body weight, hyperglycemia, and hypercholesterolemia in obese and diabetic subjects. Thus, dietary fructans have beneficial health effects in inhibiting risk factors linked to liver steatosis in diabetic patients [131].

The effect of chicory seeds was also studied in glucose tolerance and the metabolic profile in diabetic rats. Late and early stages of T2D were induced in murine models using STZ and a mix of STZ and niacinamide. The aqueous extract of chicory seeds was administered 28 days through injections of 125 mg/kg body weight. The study’s results indicated that chicory had a noticeable impact on inhibiting the progression of T2D and in hindering the evolution of associated complications [132].

The effect of *C. intybus* leaves over hyperglycemia was also studied in relation to other plants of Egyptian origin in nicotinamide-streptozotocin–induced diabetic mice. The hydroethanolic extract of *Cichorium intybus* L., as in the case of *Cassia acutifolia*, *Salix aegyptica*, and *Eucalyptus globulus* extracts, indicated the greatest anti-hyperglycemic action compared to the other examined plants, which is an action that was associated with a potential improvement of serum insulin levels [133].

Chicory seeds were evaluated regarding their influence over postprandial blood glucose, fasting blood glucose, and glycosylated/glycated hemoglobin in T2D patients (19 men and 11 women) with poor glycaemic control. The results showed that chicory seed effectively decrease fasting and postprandial blood glucose of T2D patients. The results also suggested that partially purified preparations of chicory seeds (seed powder decoction) are more valuable than crude chicory seed powder [130].

Chicory leaves were also studied for their properties in preventing diet-related T2D. In 2016, a methanolic extract was proven to inhibit interleukin secretion through the mitigation of NLRP3 inflammasome activation in T2D induced in mice through highly lipidic diets. The anti-diabetic effect was expressed by enhancing glucose metabolism and impeding meta-inflammation, which is a result triggered most likely by the plant’s content of sesquiterpene lactones such as lactucin [134].

**Figure 7:**
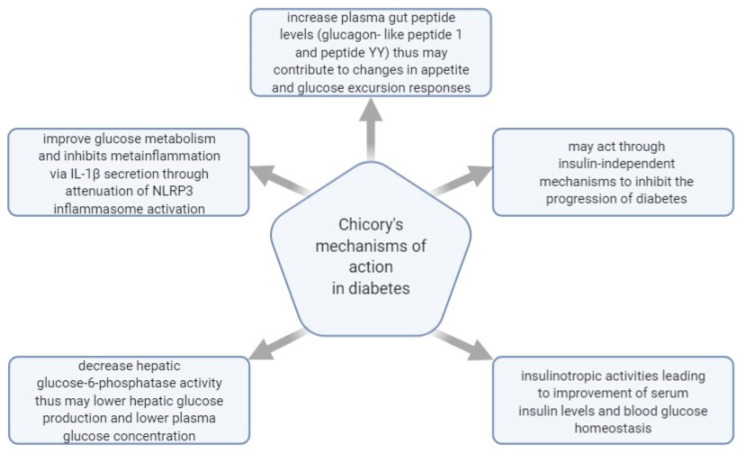
Mechanisms of action regarding chicory’s effect in managing diabetes.

Figure 7 shows some of the mechanisms of actions investigated regarding *C. intybus*’s effect in managing diabetes.

### 4.2. Chicory Extracts and Their Natural Products Studied for Therapeutic Potential in Hepatic Disorders

Although there are quite a few indications about chicory being beneficial in diabetes, there are some studies conducted over the last 10 years on chicory’s potential of managing hepatic diseases. In Table 8, there are some summaries of the studies conducted to assess the chicory’s action in managing hepatic disorders.

Chicory seeds were studied for their effect on diabetes and oleic acid-induced NAFLD and NASH. The aqueous extract of chicory seeds (containing caffeic acid, chlorogenic acid, and chicoric acid) was able to simultaneously target hyperglycemia, hyperlipidemia, insulin resistance, NAFLD, and NASH, possibly via modulation of peroxisome proliferator-activated receptor alpha and sterol regulatory element-binding protein-1c ratio [136].

To further investigate its hepatoprotective actions, *C. intybus* was studied for its ability to modulate the expression of liver cytoskeletal proteins in growing pigs. It was shown that the dried chicory root and chicory inulin acts as hepatoprotective due to their ability to regulate hepatic protein expression responsible for energetic metabolism. Moreover, having an impact on lipid-lowering mechanisms, chicory’s compounds like inulin can help manage other metabolic disorders like cardiovascular disease and diabetes. However, it was suggested that further clinical studies are needed to validate this potential [135].

Isolated polysaccharides from chicory root, predominantly comprised of sorbin, glucose, fructose, and glucitol, were studied in high-fat diet-induced NAFLD. After histopathological examination of rat livers, it was concluded that chicory polysaccharides might be efficient in managing non-alcoholic fatty liver disease via AMP-activated protein kinase [137].

The whole plant was studied as well for its action in thioacetamide-induced liver cirrhosis. Diet-supplementation of chicory powder has shown that it can protect against thioacetamide-induced liver damage, fibrosis, and cirrhosis by reducing oxidative stress and disrupting the inflammatory pathway via AMPK/SIRT1/FXR signaling [138].

**Figure 8:**
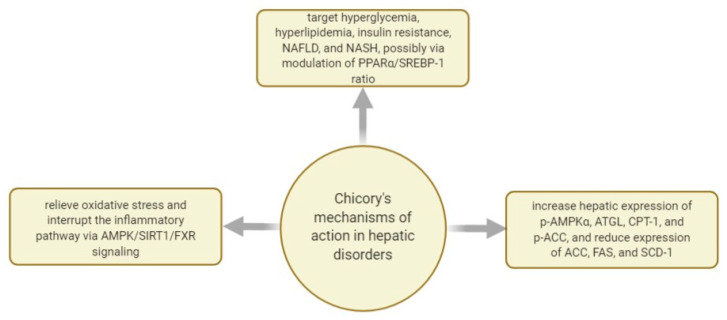
Mechanisms of action regarding chicory’s effect in managing hepatic disorders.

In Figure 8, there are some mechanisms of actions investigated regarding *C. intybus*’s effect in managing hepatic disorders.

### 4.3. Chicory Extracts and Their Natural Products Evaluated for Potential Therapeutic Potential in Cardiovascular Disease

As seen in Table 9, after checking the literature published before 2010, it was found that there are only a couple of relevant studies regarding *C. intybus* roots extracts and their potential benefits in managing cardiovascular diseases. However, in Table 9, there are some summaries of the studies conducted to assess the chicory’s action in managing cardiovascular disease.

Over the last 10 years, only a few studies indicated the chicory’s therapeutic value in managing cardiovascular diseases. For example, in 2011, a group of researchers investigated the thrombosis preventive potential of chicory coffee consumption. To study the potential cardiovascular benefits, 27 healthy volunteers drank 300 mL of coffee in the morning, made from 20 g of ground chicory coffee, for 7 days. As a result, blood and plasma viscosity were substantially diminished. Major improvements were noticed in red blood cell deformability with an overall antithrombotic and anti-inflammatory action, which might be attributed to the chicory coffee’s phenolics, including caffeic acid [139].

In another instance, the whole chicory plant was administered as a diet supplement to Triton WR-1339-induced hyperlipidemia in doses of 10 g/100 g diet for 4 weeks, and it was concluded that chicory acts as a hypolipidemic, anti-lipotoxic, antioxidant, and anti-atherogenic factor. Moreover, it was suggested that, due to its power to regulate cholesterol biosynthesis, lipogenic enzyme ACC, vascular inflammation, and redox status, *C. intybus* could protect against cardiovascular and hepatic steatosis [141].

It has been proven that an elevated level of uric acid is an independent risk factor for metabolic syndrome, cardiovascular disease, and kidney disease. In this sense, a recent study was published investigating chicory extracts’ power to ameliorate hyperuricemia in quail. Quails suffering from hyperuricemia induced by high-purine diets were fed with chicory extract at various doses. It was found that the chicory water-extract reinstated gut microbiota diversity in quail models of hyperuricemia and presented anti-hyperuricemia action, indicating that non-starch polysaccharides can enhance microbial diversity. Moreover, high (16.7 g/kg chicory inulin water solution) and middle doses (6.6 g/kg chicory inulin water solution) of chicory demonstrated a steady action of lowering serum uric acid in purine-induced hyperuricemia quails. Given this, it is suggested that chicory extracts can help reduce risk factors of CVD [140].

**Figure 9:**
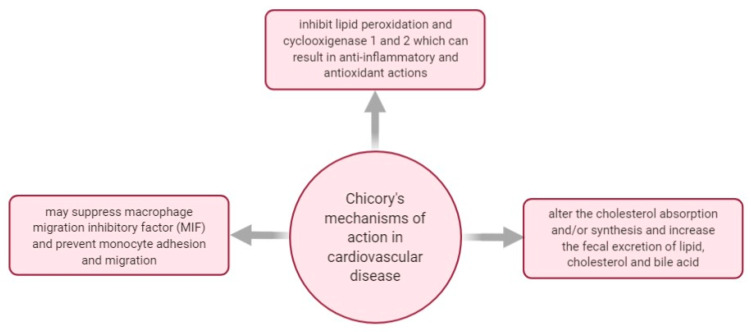
Mechanisms of action regarding chicory’s effect in managing cardiovascular disease.

In Figure 9, there are some mechanisms of actions investigated regarding *C. intybus*’s effect in managing cardiovascular disease.

## 5. Discussion

This review mainly investigated the research results published regarding various extracts of *T. officinale* leaves and roots, *M. nigra* leaves and fruits, and *C. intybus* leaves, seeds, and roots and their beneficial actions in managing diabetes, hepatic disorders, and cardiovascular disease.

Looking at the results presented in chapter 2, over the last 10 years, compared with the other parts of the same plant, dandelion leaves seemed to receive increased attention. They were intensively studied in what concerns their action in hepatic diseases, diabetes, and cardiovascular disease. The highlighted therapeutic actions of *Taraxacum officinale* L. leaves are mainly attributed to their content of natural products such as polyphenols, flavonoids, tannins, and sesquiterpenes. Thus, extracts of dandelion leaves were reported to have hepatoprotective properties in liver cancer [46], lead-poisoning [67], drug-induced hepatic damage [59,65], and non-alcoholic fatty liver disease [68,69]. However, other studies indicate that aqueous extracts of dandelion leaves are also beneficial in managing diabetes by improving carbohydrate metabolism and having α-amylase and α-glucosidase inhibitory activities [51,55]. In what concerns cardiovascular disease, dandelion root extracts were reported to have anti-platelet, hypolipidemic, and antioxidant action, which are beneficial in managing cardiovascular diseases [77,82,83]. Moreover, although this paper covered *T. officinale* extracts and their beneficial extracts and compounds in diabetes, hepatic disorders, and cardiovascular disease, water extracts of white mulberry and *Taraxacum coreanum* Nakai flower also have the potential to alleviate pathologies linked to excess alcohol consumption, as it was shown that they increase ethanol degradation, improve glucose metabolism, and ease gut microbiome dysbiosis [144]. Although not covered in this paper, *Taraxacum mongolicum* Hand.-Mazz. also has the potential of improving T2D due to its hypoglycemic effect, as seen in a study conducted on its chloroform extract in L6 cells [145].

In what concerns the *Morus* species, it seems like black mulberry leaves and some of their natural products are mainly proven to be effective in managing diabetes due to their antioxidant action [97,101] and reported the capacity of increasing a serum insulin level [100,107,110]. These therapeutic properties in managing diabetes are thought to be attributed to mulberry leaves’ compounds such as 1-deoxynojirimycin, chlorogenic acid, quercetin, and polysaccharides. In hepatic ailments, black mulberry leaves present antioxidant action [113] and cytoprotective characteristics [85], have a hypolipidemic effect, and are beneficial in dyslipidemia due to compounds such as gallic and chlorogenic acid, rutin and catechin, quercetin, luteolin, and isorhamnetin [109,113,115]. In cardiovascular diseases and associated disorders, white mulberry and black mulberry fruits are studied more than the leaves because anthocyanins present in mulberry fruits are already validated for their beneficial action in cardiovascular disease prevention [146]. Nevertheless, anthocyanins often interact with other phytochemicals, showing synergistic biological effects while making contributions from individual components that are difficult to decipher [147].

According to the reviewed publications, it seems like chicory roots were the more intensively studied plant part of chicory regarding the activity of its natural products in diabetes, hepatic disease, and cardiovascular diseases. It was shown that chicory root extracts comprising inulin and inulin-type fructans are beneficial in the management of metabolic disorders such as CVD and diabetes [135]. Chicory root polysaccharides can exert hepatoprotective and preventive actions in NASH [137], can control hyperglycemia [131], and present antithrombotic and anti-inflammatory action due to the chicory root’s phenolics like caffeic acid [139]. A few studies conducted on chicory leaves extracts suggest that chicory leaves are efficient in managing diabetes due to their ability to improve glucose metabolism and the serum insulin level [133,134]. On the other hand, chicory seeds present therapeutic benefits in diet and drug-related hepatic ailments and diabetes [130,132,136].

As previously reported, dandelion, chicory, and mulberry are medicinal plants containing significant sources of natural products with potential pharmacological applications in managing various ailments. Given their bioactive compounds, dandelion, mulberry, and chicory are potential candidates for pharmacological applications in the management of diabetes, hepatic ailments, and cardiovascular disease. Nevertheless, these plants can be used to develop functional products meant to prevent and alleviate chronic and degenerative diseases.

According to existing literature, it is known that *T. officinale, M. nigra*, and *C. intybus* present beneficial potential in managing diabetes, hepatic disorders, and cardiovascular disease. Other plants, such as *Olea europaea*, *Taraxacum campylodes*, *Urtica dioica*, *Vaccinium myrtillus*, *Acacia catechu*, *Allium sativum*, *Aloe vera*, *Cinnamomum zeylanicum*, *Gymnema sylvestre*, and *Zingiber officinale* can have, among other benefits, antidiabetic potency and, thus, can be looked at as alternative sources of future therapies [148]. As mentioned previously, *T. officinale*, *Morus nigra*, and *Cichorium intybus* are among the plants with long-standing traditional usage, and, given the available scientific findings, these too should receive further scientific interest.

Finally, transferring valuable bioactive compounds and natural products from plant matrices into functional products can help manage public health concerns [149,150,151]. Given today’s struggle to develop nutritious food with beneficial actions on one’s health status, various studies illustrate ways to obtain functional beverages using plants with high phenolic content and antioxidant activity. These biotechnologies would include fermentation processes [152,153,154].

Dandelion leaves and roots, mulberry leaves, and chicory roots seem to be studied more in-depth over the last 10 years than their other plant parts, at least in direct connection with their effect on managing or preventing diabetes, hepatic ailments, and cardiovascular disease.

Due to this, exploring the possibilities of extracting these medicinal plants’ natural products into functional foods with elevated contents of bioactive ingredients is recommended by using environmentally-friendly and cost-efficient methods. Moreover, given their availability and frequency in Europe, it is recommended to explore these plants more in-depth to further asses their therapeutic potential and their limitations and contraindications.

Given the findings of in vitro and in vivo studies published over recent years, dandelion, chicory, and mulberry present promising therapeutic benefits and are considered potential aids in managing liver diseases, diabetes, cardiovascular diseases, hepatotoxicity, and obesity. Moreover, given the intrinsic link between these medical conditions, it would also be worth to further study the interaction and synergic actions of natural products of dandelion, chicory, and mulberry extracts in the view of obtaining a final potent extract or functional product meant to prevent or alleviate degenerative diseases through hepatoprotective, antioxidant, anti-diabetic, hypolipidemic, and anti-inflammatory properties. After such a product is obtained, additional efforts should be put toward conducting clinical trials on a therapy outcome. Literature shows that such assessments are scarce and would be needed to further validate their beneficial potential.

## Figures and Tables

**Table 1:** Dandelion extracts in diabetes and their potentially responsible effective compounds.

Extract Type and Plant Part	Type of Study	Results	Responsible Functional Natural Products	References
Dandelion water extract	in vivo, *s*treptozotocin (STZ) -induced diabetic rats	- improve lipid metabolism, prevents diabetic complication resulted from lipid peroxidation and free radicals	NA	[49]
Extract P-9801091 (*Taraxaci radix* 9.7% and other medicinal plant extracts among which *Cichorii radix* 17.7% and *Mori folium* 7.4%)	in vivo, in non-obese diabetic mice	- influence lipid peroxidation and increased antioxidant action of Glutathione S-transferases in the liver, likely via a reduction in hyperglycemia	NA	[50]
Dandelion leaves and roots aqueous and ethanolic extracts	in vivo, in STZ-induced diabetes in rats	- enhance carbohydrate metabolism	- fructo-oligosaccharides (inulin)	[51]
Dandelion leaves extract	in vivo, in rat models of NAFLD	- decrease insulin resistance, may prevent NAFLD associated disorders such as diabetes	- polyphenols, flavonoids	[52]
Dandelion whole plant dried ethanolic extract (40 µg/mL)	in vitro, in insulin secretagogue activity INS-1 cells	- insulinotropic activity, potent antidiabetic action through hypoglycemic effect	NA	[53]
Dandelion water extract	in vitro, on a-glucosidase from baker’s yeast, rabbit liver, and small intestine	- potent alpha-glucosidase inhibitory activity along other traditional medicinal plant extracts	- unspecified phenol	[54]
Dandelion methanol and water extracts	in vitro, in carbohydrate metabolizing enzymes	- reduce carbohydrate enzymes activity (α-amylase and α-glucosidase)	- unspecified fructo-oligosaccharides	[55]
Dandelion leaves and stem ethanolic and aqueous extracts	in vitro, in 3T3-L1 cell culture	- suppress advanced glycation end products formation	- polyphenols	[56]

**Table 2:** Dandelion extracts in hepatic disorders and their potentially responsible effective compounds.

Extract Type and Plant Part	Type of Study	Results	Responsible Functional Natural Products	References
Aqueous extract of dandelion whole plant, *Vitis vinifera*, *Schizandra chinensis*, *Gardenia jasminoides*, *Angelica acutiloba*, and *Paeonia japonica*	in vivo, alcohol-induced hepatotoxicity in male Sprague Dawley mice	- lessen triglycerides, free fatty acids, and total cholesterol (TC) in the serum and liver, beneficial in fatty liver hepatotoxicity caused by chronic alcohol consumption	NA	[58]
Dandelion leaves hydroalcoholic extract	in vivo, in rat models of acetaminophen (APAP)-induced hepatotoxicity	- hepatoprotective impact, diminishes hepatic dysfunction induced by APA	- phenolic compounds	[59]
Dandelion whole plant extract (polysaccharide fractions)	in vivo, in rat models of CCl4-induced hepatic damage	- hepatoprotective action by regulating inflammatory responses and oxidative stress	- polysaccharides	[60]
Dandelion root ethanolic extracts	in vivo, in rat models of CCL_4_-induced hepatotoxicity	- attenuated hepatotoxicity as it reduced levels of (alanine aminotransferase) ALT, (aspartate transaminase) AST, (alkaline phosphatase) ALP, and total bilirubin	- sesquiterpene lactones	[61]
Dandelion root hydroethanolic extract	in vivo, in CCl_4_-induced hepatic fibrosis on male BALB/c mice	- reduced hepatic fibrinous deposits and reinstated histological architecture	- chlorogenic acid and other polyphenolic compounds	[62]
Dandelion leaves aqueous extract	in vivo, in CCl_4_-induced hepatitis in Sprague-Dawley rats	- decrease of oxidative stress and inflammatory processes	- luteolin, luteolin-7-O-glucoside, and polyphenols	[63]
Dandelion ethanolic and n-hexane leaves extract	in vivo, in rat models of CCL_4_-induced hepatotoxicity	- decrease thiobarbituric acid reactive substances, hydrogen peroxide and nitrite contents	- unspecified polyphenols, flavonoids	[64]
Dandelion leaves water extract	- in vivo, in rat models of sodium dichromate-induced liver damage	- antioxidant action with positive effect on alleviating hepatotoxicity and genotoxicity	- polyphenols, flavonoids, tannins, and ascorbic acid	[65]
Dandelion root extract	in vivo, in murine model of APAP-stimulated liver damage	- hepatoprotective, activating the Nrf2-Keap1 pathway	- glucose, galactose, arabinose, rhamnose, and galacturonic acid	[66]
Dandelion root extract (microspheres)	in vivo, in lead-induced hepatic damage in rats	- protects offspring from lead poisoning	- terpenes and phenolic compounds such as flavonoids	[67]
Dandelion leaves extract	in vivo, on murine models of high fat diet- induced hepatic steatosis	- reduce hepatic lipid accumulation	- luteolin and chlorogenic acid	[68]
Dandelion leaves extract	in vivo, on murine models of hepatic steatosis induced by choline and methionine deficient diet	- hepatoprotective due to antioxidant and anti-inflammatory actions	- luteolin and polyphenols	[69]
Dandelion root water extract	in vivo, on mice and in vitro, on HepG2 following ethanol exposure	- hepatoprotective action of alcohol-exposed cells and mice	- luteolin and other flavonoids	[70]
Lyophilized extract of dandelion roots and leaves	in vitro, on rat liver microsomal fraction	- reduced the enzymatically stimulated lipid peroxidation, can protect the structure of membranes, and prevent necrotizing processes	- antioxidant compounds (flavonoids)	[71]
Herbal tea mixture (peppermint, chamomile, dandelion)	in vitro, on the activity of hepatic enzymes using rat liver microsomes	- significantly inhibited some CYP isoforms, can cause alteration of phase I and II drug metabolizing enzymes	- antioxidant compounds (polyphenols and flavonoids)	[72]
Dandelion whole plant aqueous extract	in vitro, human hepatoma cell line, HepG2	- inhibited tumor cell growth, substantially induced cell death of human hepatoma cells, triggered up regulation of TNF-a and IL-1a, being potentially useful in cancer therapies	- terpenoid and sterol bitter principles (taraxasterol)	[73]

**Table 3:** Dandelion extracts in cardiovascular disease and their potentially responsible effective compounds.

Extract Type and Plant Part	Type of Study	Results	Responsible Functional Natural Products	References
Dandelion root ethanolic extracts	in vivo, on platelet anti-aggregating activity	- dose-dependent inhibition of the adenosine diphosphate -induced aggregation (40 mg dried root/mL of human platelet-rich plasma)	- polysaccharides, triterpenes, steroids	[75]
Leafy vegetable mix (12.5% each: beet leaf, angelica, red leaf lettuce, dandelion, green cos lettuce, lollo rosso, romaine lettuce, 6.25% each- scotch kale, red kale)	in vivo, in C57BL = 6 J mice on a high fat and cholesterol diet	- improved antioxidants (glutathione and b-carotene) and antioxidant enzyme activities (glutathione peroxidase, glutathione reductase, and superoxide dismutase), protects cells against lipid peroxidation and oxidative DNA damage	- polyphenolic contents	[76]
Dandelion roots and leaves	in vivo, in rabbits on a high-cholesterol diet	- may protect against atherosclerosis related to increased oxidative stress	- phenolics (catechol, caffeic acid, ferulic acid, m-coumaric acid, p-coumaric acid, vanillic acid, and syringic acid)	[77]
Dandelion leaves and petals	in vivo, in male Wistar rats	- antioxidant action, diminish triglycerides, TC, lipoprotein, and plasma atherogenic index	- L-chicoric acid	[78]
Dandelion flower extract	in vitro, in both biological and chemical models on antioxidant activity and lipid oxidation	- suppressed reactive oxygen species and nitric oxide, prevented lipid oxidation	- phenolic contents (flavonoids and coumaric acids)	[79]
Dandelion leaves, petals, and root phenolic fractions	in vitro, in human plasma, and blood platelets	- antioxidant properties, anti-platelet, and anticoagulant actions	- luteolin, cinnamic acid, L-chicoric acid, amino acid- sesquiterpene adducts	[80,81,82]
Dandelion root fractions	in vitro, in human platelets’ model	- protective action against lipids and proteins oxidation of platelets, anti-platelet action	- SL-amino acid adducts, hydroxyphenylacaetate inositol esters	[83]
Dandelion leaves and petals fractions	in vitro, in human plasma, and blood platelets	- antioxidant and anti-adhesive action	- chicoric acid	[40]

**Table 4:** Mulberry in diabetes and their potentially responsible effective compounds.

Extract Type and Plant Part	Type of Study	Results	Responsible Functional Natural Products	References
Mulberry leaves decoction extract (5%, 10%, 15%)	in vivo, 38 women, 12 men; 25–60 years old, diabetes mellitus	- reduced diabetic symptoms and eliminated neuropathy, however, did not reduce fasting blood sugars	NA	[94]
Mulberry leaves extract enriched with 1-deoxynojirimycin	in vivo, 76 human subjects with impaired glucose metabolism	- enhanced postprandial glycemic control	- polyphenols, 1-deoxynojirimycin	[95]
Extract P-9801091 (mulberry leaves and other plant extracts among *T. officinale* root and *C. intybus* root)	in vivo, on non-obese diabetic mice	- influenced lipid peroxidation and increased the antioxidative action of Glutathione S-transferases in liver, probably via a reduction in hyperglycemia	NA	[50]
Mulberry leaves extract (powder)	in vivo, in STZ–induced diabetes in rats	- suppress blood sugar levels	NA	[96]
Mulberry leaves aqueous extract	in vivo, in STZ-induced diabetic and non-diabetic Wistar rats	- antioxidant action and reduced occurrence of skeletal and visceral abnormalities	NA	[97]
Mulberry leaves ethanolic extract	in vivo, in diabetes rat models induced by high-fat diet and STZ	- antioxidant action, modulate fasting blood glucose	- 1-deoxynojirimycin, flavonoids, polyphenols, and polysaccharides	[98,99]
Mulberry leaves fractions	in vivo, in alloxan-induced diabetic mice	- reduce postprandial blood glucose and alleviate toxicity	- hybrid of 1-deoxynojirimycin and polysaccharides	[100]
Mulberry leaves extract	in vivo, STZ-induced diabetic murine model	- boost endogenous insulin secretion, have antioxidant action	- quercitrin	[101]
Mulberry leaves Hex-Mn fraction	in vivo, in diabetic mice models	- delay of carbohydrate digestion	- isoquercitrin and kaempferol-3-O-rhamnoside	[102]
Fresh and freeze-dried mulberry leaves	in vitro, on inhibition of alpha-glucosidase	- lowered blood glucose, inhibited alpha-glucosidase	NA	[103]
Mulberry (n-hexane, chloroform, ethyl acetate fractions)	in vitro, on starch breakdown of alpha-amylase activity	- dose-dependent inhibitory effects on alpha-amylase activity [IC50 = 13.26 (12.86–13.66) mg/mL]	NA	[104]
Mulberry leaves fractions	in vitro, in Caco-2 cell lines	- impede glucose absorption	- chlorogenic acid, rutin, benzoic acid, and hyperoside	[105]

**Table 5:** Mulberry extracts in hepatic disorders and their potentially responsible effective compounds.

Extract Type and Plant Part	Type of Study	Results	Responsible Functional Natural Products	References
Mulberry leaves MeOH-H_2_O extract	in vivo, paracetamol-induced hepatic lesions in rats	- hepatoprotective action	- quercetin, luteolin, and isorhamnetin	[109]
Mulberry leaves ethanolic extract	in vivo, methotrexate-induced hepatotoxicity in rats, in vitro, in HepG2 cells	- antioxidant and cytoprotective action	- flavonoids	[85]
Mulberry hydroalcoholic leaves extract	in vivo, in alloxan-induced diabetic rats	- attenuate levels of liver damage markers, hepatoprotective	- caffeoylquinic acids	[110]
Mulberry leaves lyophilized extracts	in vivo, in CCI_4_-induced hepatic injury in rats	- antioxidant action, controls the activity of SOD and GPx	NA	[111]
Mulberry leaves EtOH extract	in vivo, in female and male rats	- protective and cholesterol-lowering actions	- quercetin, caffeic acid	[112]
Mulberry leaves MeOH extract	in vivo, in rat models of APAP-induced liver damage	- genetic protection and for the treatment of organotoxicity	- polyphenols and flavonoids (gallic acid, chlorogenic acid, catechin, rutin)	[113]

**Table 6:** Mulberry extracts in cardiovascular disease and their potentially responsible effective compounds.

Extract Type and Plant Part	Type of Study	Results	Responsible Functional Natural Products	References
Mulberry leaves extract	in vivo, in hyperlipidemic murine models	- reduce TC and low-density lipids (LDL )serum levels	NA	[114]
Mulberry leaves aqueous extracts (infusion and decoction)	in vivo, in hyperlipidemic murine models	- hypolipidemic and antioxidant action, beneficial in dyslipidemia	- quercetin, chlorogenic acid	[115]
Fresh mulberry leaves (integrated in diet)	in vivo, in swine fattening models	- obesity preventive action	NA	[116]

**Table 7:** Chicory extracts in diabetes and their potentially responsible effective compounds.

Extract Type and Plant Part	Type of Study	Results	Responsible Functional Natural Products	References
Chicory seeds powder decoction	in vivo, in T2D patients (19 men and 11 women)	- decrease fasting and postprandial blood glucose	- caffeic acid, chlorogenic acid, chicoric acid	[130]
Chicory root fructans	in vivo, in normal, and obese diabetic and non-diabetic murine models	- modulate body weight, hyperglycemia, and hypercholesterolemia	- inulin-type fructans	[131]
Chicory seeds aqueous extract	in vivo, in murine diabetes models	- inhibit the progression of diabetes	- unspecified antioxidant compounds	[132]
Chicory leaves hydroethanolic extract	in vivo, in nicotinamide-STZ–induced diabetes in mice	- anti-hyperglycemic action, improve serum insulin levels	- lactucin	[133]
Chicory leaves methanolic extract	in vivo, in high-fat diet-induced diabetes in mice	- enhance glucose metabolism and impede meta-inflammation	sesquiterpene lactones (lactucin)	[134]

**Table 8:** Chicory extracts in hepatic disorders and their potentially responsible effective compounds.

Extract Type and Plant Part	Type of Study	Results	Responsible Functional Natural Products	References
Dried chicory root	in vivo, in growing swine models	- hepatoprotective action, potential therapeutic action in metabolic disorders	- inulin	[135]
Chicory seeds aqueous extract	in vivo, in diabetes, and oleic acid-induced NAFLD and NASH	- alleviate hyperglycemia, reduce lipidemic content, modulate insulin resistance, alleviate NAFLD and NASH	- caffeic acid, chlorogenic acid, and chicoric acid	[136]
Chicory root fractions	in vivo, in high-fat-induced NAFLD murine models	- alleviate NAFLD via AMP-activated protein kinase	- polysaccharides (sorbin, glucose, fructose, and glucitol)	[137]
Whole chicory plant powder extract	in vivo, in thioacetamide-induced liver cirrhosis in murine models	- reduce oxidative stress, hepatoprotective action	phenolics, chicoric acid, inulin	[138]

**Table 9:** Chicory extracts in cardiovascular diseases and their potentially responsible effective compounds.

Extract Type and Plant Part	Type of Study	Results	Responsible Functional Natural Products	References
Chicory root coffee	in vivo, in 27 healthy human volunteers	- anti-thrombotic and anti-inflammatory action	- phenolics, caffeic acid	[139]
Whole chicory water extract	in vivo, hyperuricemia induced in quail models	- reduce risk factors of CVD by lowering serum uric acid, reinstate gut microbiota diversity	NA	[140]
Whole chicory plant	in vivo, in hyperlipidemic murine models	- hypolipidemic, anti-lipotoxic, antioxidant, and anti-atherogenic action	- inulin, unsaturated sterols, flavonoids, polyphenol, and tannins	[141]
Chicory roots water-soluble extract	in vivo, on male Sprague-Dawley rats and their lipid metabolism	- lead to higher serum high density lipoprotein cholesterol and generally lower low density lipoprotein cholesterol concentrations	- inulin	[142]
Chicory leaves water extract	in vitro, lipid peroxidation (LPO) and cyclooxygenase (COX-1 and COX-2) enzyme inhibitory activities	- inhibited LPO and COX- 1 and COX-2, which can result in anti-inflammatory and antioxidant actions	- anthocyanins (cyanidin-3-O-glucoside)	[143]

## Data Availability

Not applicable.
